# Risk Factors for Anthroponotic Cutaneous Leishmaniasis at the Household Level in Kabul, Afghanistan

**DOI:** 10.1371/journal.pntd.0000639

**Published:** 2010-03-23

**Authors:** Richard Reithinger, Mohammad Mohsen, Toby Leslie

**Affiliations:** HealthNet TPO, Kabul, Afghanistan; Institute of Tropical Medicine, Belgium

## Abstract

**Background:**

Kabul, Afghanistan, is the largest focus of anthroponotic cutaneous leishmaniasis (ACL) in the world. ACL is a protozoan disease transmitted to humans by the bite of phlebotomine sand flies. Although not fatal, ACL can lead to considerable stigmatization of affected populations.

**Methods:**

Using data from a standardized survey of 872 households in 4 wards of Kabul, Afghanistan, univariate and multivariate logistic regression analyses tested associations between presence of active ACL and ACL scars with 15 household-level variables.

**Findings:**

Univariate analyses showed that active ACL was positively associated with household member's age, ACL prevalence, and brick wall type, but negatively associated with household number of rooms, bednet use, and proportion of windows with screens. Multivariate analysis showed a positive association between active ACL and household member's age, ACL prevalence, and brick wall type, and a negative association with household proportion of windows with screens.

**Conclusion:**

Household-level charateristics were shown to be risk factors for ACL. Monitoring a selected number of household characteristics could assist in rapid assessments of household-level variation in risk of ACL. ACL prevention and control programs should consider improving house construction, including smoothing of walls and screening of windows.

## Author Summary

Cutaneous leishmaniasis is a vector-borne protozoan disease that is characterized by cutaneous lesions which develop at the site of the insect bite. Lesions can vary in severity, clinical appearance, and time to cure; in a proportion of patients lesions can become chronic, leading to disfiguring mucosal leishmaniasis or leishmaniasis recidvans. Albeit not fatal, cutaneous leishmaniasis can have a significant social impact as it may lead to severe stigmatisation of affected individuals when lesions or scars occur on the face and exposed extremeties. Over the last 10–20 years there has been an increase in the number of leishmaniasis cases reported in South Asia, particularly in Afghanistan. Little is known about the household-level risk factors for infection and disease. Here we confirm previous reports that had shown the association of cutaneous leishmaniasis with age and clustering of cases at the household-level. Additionally, we show that risk of cutaneous leishmaniasis is associated with household construction (i.e. brick walls) and design (i.e. proportion of windows with screens).

## Introduction

After decades of war, Kabul, Afghanistan, became the world's largest focus of cutaneous leishmaniasis (CL) with an estimated annual incidence of 67,500 cases [1]. CL is a vector-borne protozoan disease that is characterized by cutaneous lesions which develop at the site of the insect bite [2]. Albeit not fatal, CL has a significant social impact in Afghanistan [3],[4] as it may lead to severe stigmatisation of affected individuals when lesions occur on the face and exposed extremeties.

Several reports document the dramatic increase of CL in the region, including Iran [5]–[7], Pakistan [8]–[11] and, in particular, Afghanistan [1], [12]–[16]. Whilst some of these reports have indicated that CL is associated with sex [1], age [1],[9], domestic animals [9] and clustering of cases at the household level [1],[9] little is known about other household characteristics associated with this disease in South Asia. Whereas is many Asian foci CL is transmitted zoonotically (i.e. through an animal reservoir), CL in Kabul is transmitted anthroponotically, with CL being referred to as anthroponotic CL (ACL) and the human population representing the main reservoir of infection.

The objectives of the work described here were to investigate household-level characteristics associated with ACL in Kabul in order to support the development and implementation of strategies to prevent and control ACL.

## Materials and Methods

### Survey Design and Methodology

The study was carried out in September 2003 in four (III, IV, VI, VII) of the city's 14 wards. These wards were purposefully selected as an ACL prevalence of >2% had been observed during a survey conducted in 2002 (Reithinger *et al., unpublished*). ACL transmission is from April to October, but cases are seen throughout the year due to the disease's long pre-patent period (up to 6 months) [2]. Wards were divided into clusters of 10 households on a map provided by the Kabul Institute of Cartography, with 25 randomly-selected clusters in each ward being included in the study. Thus, the total sample was 1,000 households, or 250 households in each ward.

Household members' demographic, clinical and household data were recorded by trained survey staff using a standardized, pre-tested questionnaire. Demographic data included information on individual household member's age and sex and household family size. For clinical data, medical staff diagnosed disease in household members on the basis of presence or absence of ACL lesions or scars, number of lesions, and date of lesion onset [1]. Because of logistic constraints and the high sensisitivity and specificity of clinical diagnosis, parasitologic diagnosis of ACL lesions (e.g. microscopic examination or parasite culture) was not carried out; most of the infections in Kabul are caused by *Leishmania tropica* [17]. All persons with active lesions were offered free anti-leishmanial treatment at any of the 8 HealthNet TPO leishmaniasis clinics in the city. Household data included information on household design (i.e. number of rooms, number of windows), construction materials (i.e. wall type, ceiling type), ACL preventive methods (i.e. number of windows screened, household bednet ownership, reported bednet use), and ownership of animals (i.e. household ownership of dogs, chicken, goats, sheep and cattle).

### Statistical Analysis

Questionnaire data was entered into a Microsoft Excel database (Microsoft Corporation, Seattle, WA). In addition to explanatory variables collected during the household surveys, a number of explanatory variables were derived, including household prevalence of active or past ACL, household members per room, number of windows per household members and proportion of household windows screened. Note, for calculation of household prevalence of active or past ACL, the household member under study was excluded from the prevalence calculation. In a first set of analyses, the association of possible household-level explanatory variables with individuals having active ACL or ACL scars was tested by estimating univariate odds ratios (OR) by logistic regression. In a second set of analyses, we used backward stepwise multiple regression to identify significant (p<0.05) explanatory variables predicting household occurrence of individuals having active ACL or ACL scars [9]. Because ACL transmission in Kabul is focal [1], we adjusted all of our analyses by individual households to provide robust standard errors. Finally, multicollinearity was assessed by testing the variance inflation factor of explanatory variables prior to their inclusion in the multivariate model [18]. Variance inflation factors >5 indicate collinearity and that variables may be redundant; such variables were dropped prior to inclusion of variables for the full multivariate model. All analyses were done with Stata software, version 9.2 (Stata, College Station, Texas).

### Ethical Statement

The data reported in the manuscript are part of a larger research study that investigated the efficacy of insecticide-treated bednets for protecting Kabul residents from ACL. Approval to conduct the study was given by the Institute of Malaria and Parasitic Diseases, Afghan Ministry of Health. In 2003, the Institue of Malaria and Parasitic Diseases did not have an Ethical Review Board; HealthNet TPO is a small non-governmental organization (NGO) that does not have an Ethical Review Board either. At the time of the study, the Afghan Ministry of Health and the entire country were still in a status of transition, with many (previously defunct) institutions being established or strengthened. As an operational NGO working in complex emergency settings our pimary goal was -whilst adhering to the tenets of the Declaration of Helsinki- to tackle the ACL epidemic that was raging in the city at that time as quickly as possible. As no biological samples were collected and because the study was part of the monitoring and evaluation process of an operational program, only verbal informed consent was obtained from study participants. Consent to participate in interviews was sought from the household heads and each eligible individual; whether consent was given or not was noted on the survey questionnaires.

## Results

### Population Surveyed and Prevalence of ACL

A total of 996 households were visited; 124 households had incomplete demographic or clinical data and were excluded from the analyses. Of the 10,596 people surveyed in the 872 households included (mean: 12.2 persons per household), 51.1% and 48.9% where male and female, respectively; the median age of the study population was 15 years (interquartile range [IQR] 8–30). Households had a mean 2.0 rooms (range 1–22), 2.2 windows (range 0–18), 4.6 household members per room (range 0.18–15) and 0.4 windows per household member (range 0–4).

**Figure 1:**
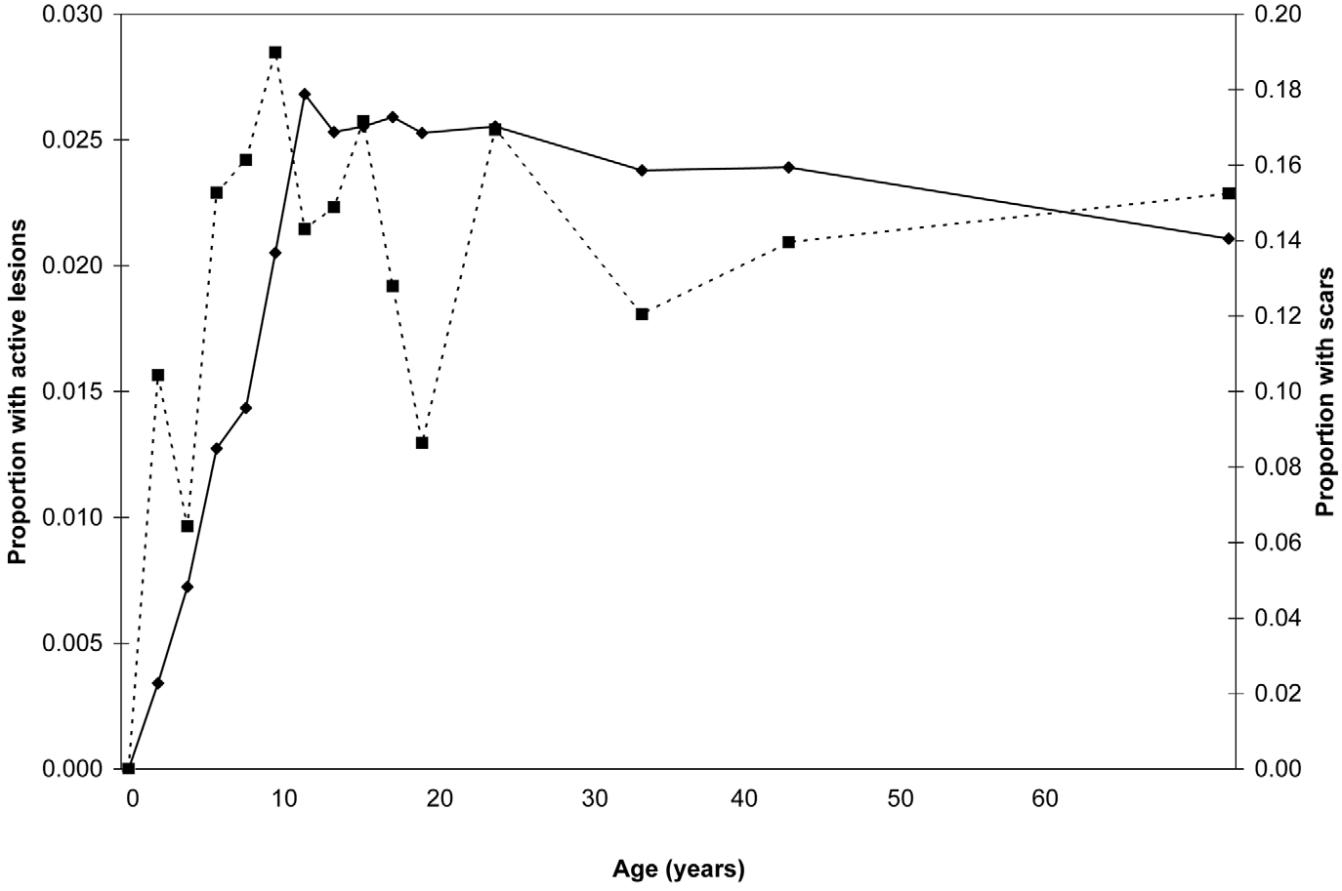
Age prevalence curve of anthroponotic cutaneous leishmaniasis (ACL) in Kabul, Afghanistan. Dotted line: active ACL lesions; continuous line: ACL scars.

Of the population surveyed, 224 (2.1%) and 1,421 (13.4%) had active ACL lesions or scars, respectively; 11 individuals had both lesions and scars (**Figure 1**). Of those persons with ACL lesions, the median lesion number was 1 (IQR: 1–2) and the median lesion duration (to survey date) was 8.5 months (IQR 0.75–48). The median age of those individuals with ACL lesions was 15 years (IQR 9–30) and of those with ACL scars was 18 (IQR 12–30) (**Figure 1**).

### Household-Level Variables: Univariate Analysis

The univariate analysis showed that whereas risk of active ACL was positively associated with household members' age, particularly age groups of 19 years of age and younger (**Figure 1**), the prevalence of active ACL, brick walls and population density, it was negatively associated with the household number of rooms and the proportion of windows that are screened (**Table 1**). Risk of ACL scars was positively associated with household member's age (**Figure 1**), prevalence of ACL scars, number of windows per household member, and brick or stone walls, but negatively associated with household wood-beamed ceilings and population density (**Table 1**).

**Table 1:** Univariate regression analysis.

Risk Factor			Cutaneous leishmaniasis lesion				Cutaneous leishmaniasis scar			
		Total	%	OR	95% CI	*P*	%	OR	95% CI	*P*
**Age (years)**	0–4	1428	1.28				3.57			
	5–9	1816	2.48	1.99	1.15–3.44	0.014	9.20	2.77	2.00–3.81	<0.001
	10–14	1781	2.30	1.84	1.03–3.30	0.039	16.96	5.60	4.06–7.72	<0.001
	15–19	1348	2.52	2.02	1.07–3.84	0.031	17.28	5.75	4.03–8.22	<0.001
	≥20	4215	2.04	1.63	0.99–2.68	0.054	16.09	5.25	3.84–7.19	<0.001
**Sex**	Male	5410	2.01				13.03			
	Female	5186	2.22	1.10	0.86–1.41	0.437	14.02	1.09	0.97–1.23	0.149
**Household active ACL prevalence**	0.00	8706	0.93				13.50			
	0.01–0.25	1786	6.55	7.45	4.78–11.63	<0.001	13.77	1.03	0.76–1.39	0.856
	>0.25	101	25.74	36.86	18.32–74.18	<0.001	10.89	0.79	0.28–2.19	0.647
**Household ACL scar prevalence**	0.00	4819	2.18				3.07			
	0.01–0.25	3943	2.18	1.00	0.63–1.58	0.996	14.71	5.44	4.11–7.21	<0.001
	>0.25	1833	1.80	0.83	0.45–1.54	0.551	38.35	19.57	15.08–25.41	<0.001
**Household wall type**	Mud	1481	2.16				8.99			
	Brick	1391	4.89	2.33	1.21–4.47	0.011	15.10	1.80	1.19–2.72	0.005
	Stone	7446	1.64	0.75	0.44–1.31	0.315	13.99	1.64	1.17–2.30	0.004
	Other	210	0.95	0.44	0.10–1.87	0.263	20.48	2.61	1.32–5.15	0.006
**Household ceiling type**	Cloth-covered	938					22.39			
	Concrete	214	1.71	1.95	0.71–5.37	0.197	11.68	0.46	0.19–1.08	0.074
	Wood (beam)	8945	2.18	1.26	0.50–3.17	0.625	12.35	0.49	0.35–0.68	<0.001
	Wood (thatched)	142	3.52	2.10	0.65–6.85	0.217	26.76	1.27	0.63–2.55	0.507
	Other	230	1.74	1.02	0.29–3.60	0.976	17.83	0.75	0.35–1.61	0.462
**Household number of rooms**	1	4316	2.66				13.88			
	2–4	5872	1.82	0.68	0.46–1.00	0.051	13.15	0.95	0.76–1.18	0.626
	>4	349	0.06	0.21	0.05–0.84	0.027	14.61	1.07	0.60–1.93	0.819
**Household members per room**	≤2.50	2055	1.41				18.00			
	2.51–5.00	5257	1.85	1.31	0.83–2.09	0.249	11.30	0.58	0.45–0.74	0.000
	5.01–7.50	1885	2.81	2.03	1.14–3.61	0.016	15.01	0.80	0.59–1.07	0.134
	>7.51	1338	3.36	2.43	1.33–4.46	0.004	13.00	0.68	0.48–0.96	0.027
**Household number of windows**	≤1	2456	1.91				14.21			
	2–4	6560	2.44	1.30	0.81–2.09	0.271	13.17	0.93	0.74–1.16	0.519
	>4	1512	1.12	0.59	0.30–1.18	0.137	14.15	1.01	0.74–1.38	0.950
**Number of windows per household members**	≤0.25	3631	2.15				13.27			
	0.26–0.50	4580	2.25	1.04	0.72–1.51	0.818	12.38	0.93	0.74–1.17	0.547
	0.51–1.00	2056	1.85	0.85	0.44–1.67	0.647	15.81	1.24	0.95–1.62	0.116
	>1.00	259	1.54	0.71	0.22–2.34	0.567	20.08	1.66	1.00–2.75	0.049
**Proportion of household windows screened**	0.00	4172	2.90				14.19			
	0.01–0.25	588	0.85	0.28	0.09–0.96	0.043	14.11	1.00	0.64–1.56	0.994
	0.26–0.50	1687	1.42	0.48	0.28–0.82	0.007	13.63	0.96	0.70–1.31	0.794
	>0.50	3510	1.62	0.55	0.35–0.87	0.010	12.62	0.87	0.69–1.10	0.242
**Household bednet ownership**	No	10365	2.13				13.52			
	Yes	211	1.42	0.69	0.23–2.01	0.493	14.22	0.94	0.45–1.95	0.862
**Household bednet use**	No	10416	1.72				16.80			
	Yes	180	1.11	0.54	0.15–1.94	0.342	14.44	0.93	0.42–2.10	0.870
**Household ownership of animals**	No	6698	2.30				13.23			
	Yes	3859	1.79	0.77	0.54–1.10	0.155	14.07	1.08	0.88–1.33	0.464
**Household ownership of dogs**	No	7166	2.16				12.96			
	Yes	3430	2.01	0.93	0.65–1.32	0.676	14.66	1.16	0.94–1.43	0.171
**Household ownership of chicken**	No	9790	2.19				13.49			
	Yes	806	1.24	0.56	0.23–1.36	0.203	15.97	1.02	0.71–1.49	0.882

Unadjusted odds ratios for variables associated with anthroponotic cutaneous leishmaniasis in Kabul, Afghanistan.

### Household-Level Variables: Multivariate Analyses

**Figure 2:**
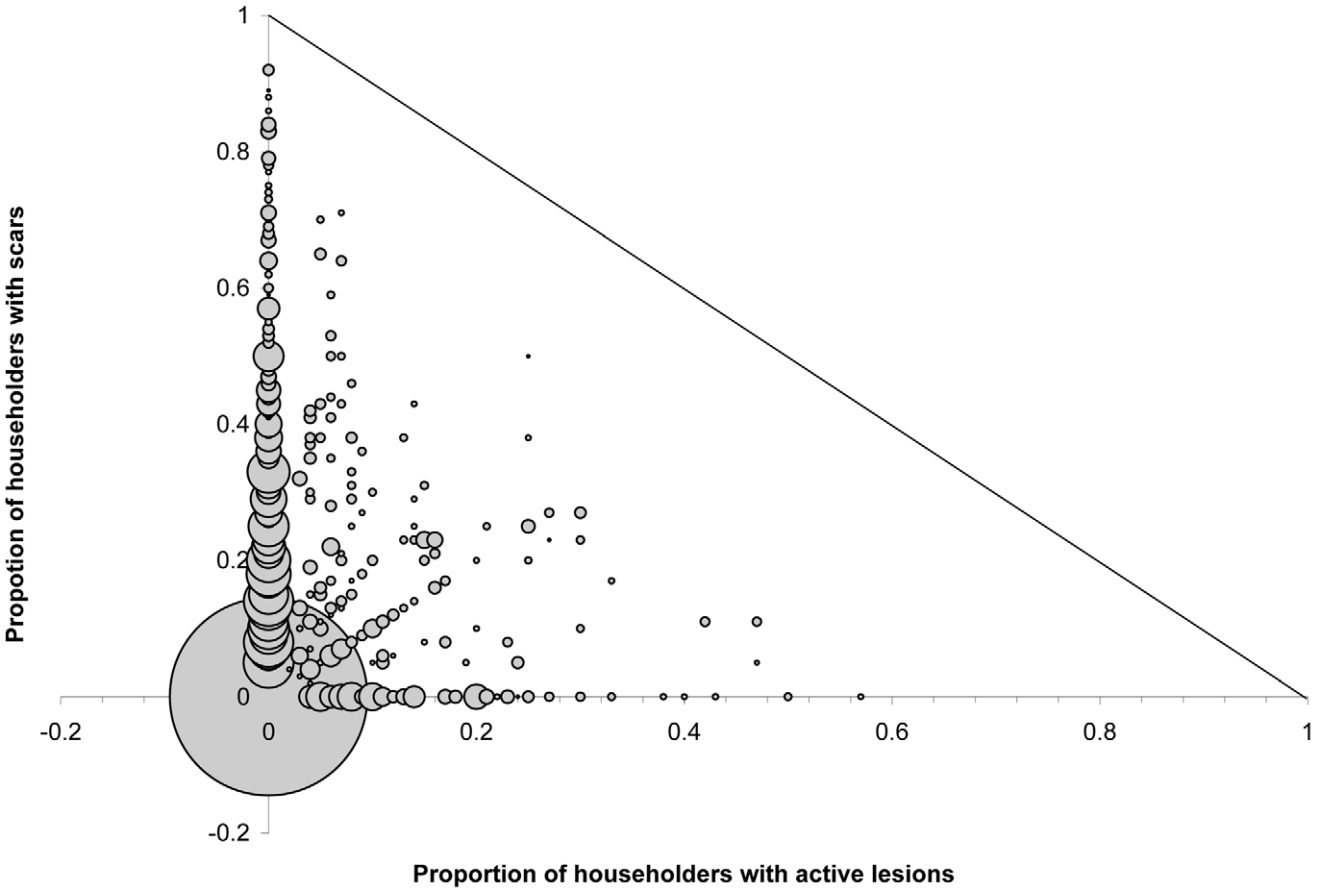
Household clustering of active and past anthroponotic cutaneous leishmaniasis (ACL) cases. Bubble graph, where the size of the data point (‘bubble’) is proportionate to the number of individuals. Straight line represents the cut-off for possible active ACL: past ACL household ratio.

Multivariate analyses demonstrated that household member's age, prevalence of active ACL (**Figure 2**) and brick walls increased the risk for active ACL, whereas the proportion of household windows screened reduced it (**Table 2**). Similarly, household member's age, prevalence of ACL scars, and brick or stone walls increased risk of ACL scars, whereas increased household prevalence of active ACL, wood-beamed ceilings and population density reduced it (**Table 2**).

**Table 2:** Multivariate regression analysis.

Risk Factor		Cutaneous leishmaniasis lesion			Cutaneous leishmaniasis scar		
		OR	95% CI	P	OR	95% CI	P
**Age (years)**	0–4						
	5–9	1.75	0.98–3.14	0.059	3.22	2.27–4.59	<0.001
	10–14	1.71	0.92–3.19	0.090	7.13	5.02–10.13	<0.001
	15–19	1.88	1.00–3.53	0.050	6.86	4.67–10.06	<0.001
	≥20	1.57	0.93–2.64	0.091	6.42	4.54–9.08	<0.001
**Household active ACL prevalence**	0.00						
	0.01–0.25	6.66	4.14–10.70		0.99	0.82–1.21	0.964
	>0.25	27.07	15.06–48.68		0.60	0.43–0.83	0.002
**Household ACL scar prevalence**	0.00						
	0.01–0.25				5.47	4.10–7.31	<0.001
	>0.25				19.91	15.16–26.15	<0.001
**Household wall type**	Mud						
	Brick	1.70	1.00–2.89	0.048	1.32	1.02–1.71	0.035
	Stone	0.99	0.62–1.61	0.983	1.24	1.01–1.52	0.039
	Other	0.28	0.04–1.85	0.185	1.50	0.84–2.68	0.172
**Household ceiling type**	Cloth-covered						
	Concrete				0.59	0.33–1.05	0.073
	Wood (beam)				0.64	0.50–0.83	0.001
	Wood (thatched)				0.85	0.53–1.36	0.504
	Other				0.82	0.48–1.39	0.458
**Household members per room**	≤2.50						
	2.51–5.00				0.70	0.58–0.83	<0.001
	5.01–7.50				0.88	0.71–1.07	0.211
	>7.51				0.71	0.54–0.94	0.017
**Proportion of household windows screened**	0.00						
	0.01–0.25	0.59	0.22–1.59	0.299			
	0.26–0.50	0.62	0.41–0.96	0.030			
	>0.50	0.80	0.59–1.10	0.166			

Note, significant collinearity was observed in the ‘Household ownership of animals’, ‘Household ownership of dogs’, ‘Household bednet ownership’ and ‘Household bednet use’ variables, with variance inflation factors of 13.55, 11.79, 6.80, 6.75, respectively. Variance inflation factors >5 indicate collinearity and that variables may be redundant. As a result the ‘Household ownership of animals’ and ‘Household bednet ownership’ variables were dropped prior to inclusion of variables for the full multivariate model.

Adjusted odds ratios for variables associated with anthroponotic cutaneous leishmaniasis in Kabul, Afghanistan.

## Discussion

In established endemic areas, ACL prevalence typically increases with age up to 15 years, after which prevalence levels off, presumably because of the acquisition of immunity [2]. Susceptibility to infection and disease is determined by a number of parasite, host and sand fly effects and factors [2]. Thus, infections can cluster within households, which is indicative of the short flight range of sand flies [19], anthroponotic transmission [1],[9], or genetic susceptibility [20]. Infection is also known to be dependent on host nutritional status and acquired immunosupression (e.g. HIV) [21],[22]. Risk factors of disease commonly include sex (e.g. sex bias usually points to behavioural patterns that increase vector exposure), age, household design and construction material (e.g. number of floors, number of rooms, dirt floor), household presence of domestic animals (e.g. dogs, pigs), household proximity to forested areas or other areas where sand flies are known to aggregate, and migration of household members [23]–[31].

Our findings indicate that household construction materials (e.g. brick walls) and design (e.g. number of rooms or number of windows per person) can significantly influence ACL risk in Kabul. Observed associations are probably due to sand fly and human behaviour, and ultimately increased or decreased sand fly exposure. Many households in surveyed city wards were still rudimentary at the time of the survey and were being re-constructed following the years of armed conflict. The sand fly vector in Kabul, *Phlebotomus sergenti*, is endophagic (i.e. biting occurs inside houses) and bites in the evening and at night [32]. Thus, for example, greater density in terms of of household members per room could attract more sand flies, increasing an individual household member's exposure to sand flies and, hence, the probability of receiving an infective bite. In contrast, an increased number of household rooms likely reduces household members' exposure to sand flies, with inside walls and doors possibly representing a barrier for host-seeking sand flies.

Our analyses showed that ACL risk is not associated with household ownership of domestic animals, specifically dogs or chicken. Although dogs have been found infected with *L. tropica* [33] and can be risk factors for ACL as reported in a study in Pakistan [9], our findings suggests that they are only incidental hosts in Kabul. This also would further confirm the anthroponotic nature of *Leishmania* transmission in Kabul. Besides dogs and chicken, people often owned cows or buffalos, sheep and goats, but these were shown to be of no significance in terms of risk for ACL (data not shown).

ACL risk is strongly associated with the presence of disease in other household members, confirming previous findings that the disease is highly focal at the household level [1]. A likely explanation for this is that sand fly distribution and abundance is patchy (both horizontally and vertically [29]), but stable over time, and –as shown here- vector exposure is strongly dependent on household construction materials and design. The sand fly flight range is generally short [19] and it is likely that *Leishmania* transmission does not occur beyond a defined cluster of households. This may also explain why risk of active disease is reduced in those households with a high proportion of household members with ACL scars; this also may explain why there is a strong association between household prevalence of active ACL and household prevalence of ACL scars (**Figure 2**). It is believed that sand flies get infected with *Leishmania* when biting people with active ACL. Thus, a household with a high proportion of people with scars is –from an epidemiological point of view- less ‘infectious’ to its inhabitants than a household that includes a high proportion of ACL cases.

Finally, our data also suggests that means to reduce vector exposure can be highly successful in reducing the risk of ACL, with simple screening of windows being effective (i.e. in this study, 45–72% protective efficacy depending on the proportion of windows screened). Use of textile fabrics, whether insecticide-treated or not, hung on doors, used as bednets or topsheets, have consistently shown efficacy in reducing indoor ACL transmission in endemic areas [34]–[36], including in Kabul [37]. Of note is that we did not observe an association of bednets or bedet use with active ACL or ACL scars, which probably was due to the small number of bednets (i.e. 211) present in surveyed households. Whether bednets are protective against *Leishmania* infection will dependent on a number of factors besides use, including whether the nets are impregnated with insecticide, net shape and size, or wear and tear [38]. Interestingly, screening of household windows and ceilings has been shown to be effective in reducing malaria vector abundance and child anemia [39]; such approach has yet to be tested for leishmaniasis.

A number of caveats have to be noted. First, we did not confirm parasite etiology of clinical cases. However, as reported previously, it is likely that most infections were due to *L. tropica* [17]. We observed clustering of cases and higher risk of ACL among younger age groups in the study, both of which are a characteristic of anthroponotic *Leishmania* transmission [1],[9]. The transmission cycle of the other *Leishmania* species causing CL in Afghanistan, *L. major*, is zoonotic, with all age groups at risk of the disease [15]. Second, we did not collect data on household members' residency time in Kabul, which should have been assessed as it could have potentially confounded the study's results. Consequently, we cannot specify whether household members did acquire ACL when living in Kabul or in another area prior to relocating to Kabul. This could have affected associations between ACL scars and household-level characteristics. However, we note that in a previous study active ACL prevalence was not shown to be significantly greater in immigrants than local Kabul residents, supporting the hypothesis that the epidemic in Kabul has been maintained by a steady influx of susceptible immigrants and that transmission is stable [13]. We also note that associations between tested household variables and ACL are remarkably similar for those with active disease and those with ACL scars. Third, we used prevalence estimates as outcome variables. These will give only a valid reflection of current transmission rates if spatial differences in transmission are constant over time. Fourth, some of the variables investigated may be a proxy indicator for other variables that were not assessed, e.g. household animal ownership and number of rooms may be a proxy for household socio-economic status and better living conditions as a whole; wood beamed households could be older and therefore in areas with less structural disruption due to the armed conflict. Fifth, the number of regression analyses that were carried out will also have increased the odds of finding a significant association between household variables and ACL. Nonetheless, we note that both univariate analyses and multivariate analyses were consistent in identifying certain household-level characteristics as being risk factors for ACL.

### Conclusion

In 2003 ten clinics diagnosed and treated leishmaniasis cases in Kabul [1], but it was estimated that only 40% (i.e. ∼25,000 cases) of all active cases were being treated annually. As ACL in Kabul is transmitted anthroponotically, this means that up to 60% of cases remained untreated and, hence, remain the main ACL reservoirs driving transmission. This may explain why the witnessed epidemic has been so prolonged since first documented in 1990 [12].

Given that Kabul is the world's largest ACL foci and given the local importance of the disease, it is recommend that large-scale strategies to reduce sand fly human vector contact, and provision of treatment be implemented. Clearly, we show that when designing an ACL intervention strategy, household variables that could represent a risk factor for infection and, therefore, could impact the intervention's success or failure should be assessed. As shown here simple measures, such as screening of windows, could significantly reduce the risk of acquiring ACL.
